# Representing the Good: Pastoral Care in a Secular Age

**DOI:** 10.1007/s11089-018-0826-0

**Published:** 2018-06-21

**Authors:** Carmen Schuhmann, Annelieke Damen

**Affiliations:** Department of Globalization and Dialogue Studies, University of Humanistic Studies, Kromme Nieuwegracht 29, 3512 HD Utrecht, the Netherlands

**Keywords:** Pastoral care, Spiritual care, Chaplaincy, ‘Secular age’, Transcendence, Iris Murdoch, Existential and spiritual processes

## Abstract

In ‘a secular age’ (Taylor 2007), pastoral care is no longer exclusively associated with specific religious traditions and communities. Pastoral caregivers who work in secular institutions provide care to religious and nonreligious people alike, and in several Western societies the term pastoral care is used in relation to nonreligious (humanist) care. In secular contexts, the term ‘pastoral care’ is often replaced by the term ‘spiritual care.’ Spiritual care, however, is provided by various professionals, so pastoral caregivers face the challenge of developing adequate and convincing language to explain what is distinctive about their work. In this article, the authors turn to philosophical language in order to develop a conceptual understanding of pastoral care that does not depend on the specific worldview—religious or nonreligious—of either pastoral caregivers or receivers of pastoral care. Using the work of Taylor (1989, 2007) and Murdoch (1970), we explain pastoral care as engaging with people’s attempts to orient in ‘moral space’ and the distinctive quality of pastoral care as ‘representing the Good.’ Murdoch associates ‘the Good’ with a secular idea of transcendence that is both a movement beyond the ego and an engagement with the reality of human vulnerability, suffering, and evil. We argue that pastoral caregivers who ‘represent the Good’ have the task not only of supporting the existential and spiritual processes of individuals but also of promoting dialogue and social justice and of critiquing dehumanizing practices in the organizations in which they work and in society at large.

Historically, the term ‘pastoral care’ has referred to care offered within Christian and Jewish communities (Doehring 2015). Over time, the term has come to designate care in the context of other religious traditions too, including the Muslim, Buddhist, and Hindu traditions. In our religiously plural world, the boundaries between religious communities are hazy since people generally draw from different religious traditions in their lives (Ammerman 2010). This challenges a narrow understanding of pastoral care as taking place within separate religious communities and traditions. These days, we live not only in a world of religious pluralism but also—at least in the West—in a secularizing world. Taylor (2007) speaks about a ‘secular age’ and explains the shift to secularity in Western societies as “a move from a society where belief in God is unchallenged and indeed, unproblematic, to one in which it is understood to be one option among others” (p. 3). This raises the question of how to understand pastoral care in a secular age. When pastoral care is associated with religion, the value of pastoral care for nonreligious people is not obvious and pastoral care may be thought of as an outdated practice. On the other hand, in various Western countries the term pastoral care no longer exclusively designates care by religious professionals but is also used in relation to nonreligious care provided by humanist practitioners.

Several authors point to the need to develop adequate and convincing language to describe the work of pastoral caregivers in a changing world (Harding et al. 2008; Kevern and McSherry 2015; Lee 2002; Thorstenson 2012). In religiously plural contexts, the term pastoral care is often dropped altogether and replaced by the term spiritual care, which is not associated with a specific religious tradition (Doehring 2015; Orton 2008; Pargament 2007). Switching to the term spiritual care has advantages in secular contexts, too. Spirituality is a complex concept that has been defined in various ways (Hill et al. 2000; de Jager Meezenbroek et al. 2012; Lephard 2015). Some of these definitions refer in the first place to personal quests for meaning by individuals (Hill et al. 2000; Walton 2012). Such descriptions do not have a religious connotation, which may have contributed to the increasing attention given to spirituality and spiritual care in, for instance, health care contexts (Lee 2002; Orton 2008; Paley 2008). However, the haziness of the term spirituality also results in unclarity concerning the question of who is entitled to provide spiritual care to whom in secular contexts. This question seems particularly urgent for chaplains who work in secular institutions and organizations such as hospitals, prisons, and the military. Chaplains have generally adopted a discourse of spirituality instead of religion to describe their work but still struggle to explain and legitimize their work (Harding et al. 2008; Lee 2002; Pesut 2016; Pesut et al. 2012). Concerns have been voiced that “chaplains are losing their authority with respect to the spiritual realm as other health care professionals claim they can and do provide spiritual care” (Harding et al. 2008, p. 115). In a secular age, chaplains face the challenge of explaining what is distinctive about the care they provide, especially when receivers of this care regard themselves as nonreligious (Nolan 2016). Some authors argue for a return to more explicitly theological instead of spiritual discourse for describing pastoral care (Harding et al. 2008), whereas others emphasize that “in a society where the proportion of people who report themselves as of no religion is increasing. .. there is need for a discourse on chaplaincy which preserves its core value but speaks to people of all religions and none” (Kevern and McSherry 2015, p. 49). Although spiritual discourse seems too general to express this “core value,” theological language does not speak to “people of all religions and none.”

In this article, we address the question of how to understand the distinctively ‘pastoral’ quality of pastoral care in a secular age. We aim for an inclusive conceptual understanding of pastoral care, both in the sense of including “people of all religions and none” as potential receivers of pastoral care and in the sense of including practitioners of various worldview traditions as potential pastoral caregivers—traditional ministers as well as humanist chaplains and indigenous caregivers. To that end, we turn to philosophical instead of explicitly theological discourse. We particularly draw from philosophical work by Taylor (1989, 2007) and Murdoch (1970), who both use terminology that, to a certain extent, reflects theological language. Their work therefore seems especially helpful in building bridges between theological and secular conceptions of pastoral care. First, we use ideas of Taylor in order to describe pastoral care as work in ‘moral space,’ elaborating on the view that pastoral caregivers engage with existential processes. We then use Murdoch’s moral philosophy in order to explain the specific pastoral quality of this engagement as ‘representing the Good.’ Here, it is important to note that, although the term ‘the Good’ may easily seem to refer to a level beyond the mundane, Murdoch’s rich, complex, and subtle understanding of the term does not separate ‘the Good’ from ordinary life. It is connected to a secular idea of transcendence that Murdoch explains as a movement beyond the ego that is simultaneously a movement towards reality. In particular, transcendence involves looking at the reality of human vulnerability, suffering, and evil. We argue that the richness of Murdoch’s concept of ‘the Good’ is helpful for specifying the pastoral character of various aspects of pastoral care practice. Pastoral caregivers who ‘represent the Good’ not only have the task of supporting the existential and spiritual processes of individuals, but they also have a role to play in the organizations in which they work and in society at large. Here, it is their task to promote dialogue, moral deliberation, and social justice and to critique oppressive or dehumanizing practices.

## Human existence and the orientation metaphor

According to Murdoch (1970), developing an understanding of the human condition necessarily involves the use of metaphorical language, in particular metaphors of space, of movement, and of vision. In several disciplines, human existence is described in terms of the spatial metaphor of orientation. From an anthropological perspective, Geertz (1973) asserts that experiencing life as meaningful requires the ability “to orient ourselves effectively within [life]” (p. 100). Psychologist Janoff-Bulman (1992), in her research on trauma, found that traumatic events shatter “basic views of ourselves and our external world that represent our orientation towards the ‘total push and pull of the cosmos’” (p. 4). Park (2010), in her psychological model of meaning-making, points to the metaphorical character of meaning-making processes and describes these as connecting life events to “general orienting systems” (p. 258).

From a philosophical perspective, Taylor (1989) has elaborated on the spatial metaphor of orientation in life in order to give a comprehensive account of human existence. Here, the metaphorical space in which processes of orientation in life take place is the space of existential questions: questions of how to live our life. Taylor refers to this metaphorical space as ‘moral space,’ “the space of questions about the good” (Taylor 1989, p. 41). It is important to note here that Taylor understands ‘moral’ and ‘morality’ in a broad and somewhat unconventional way:

In fact, I want to consider a gamut of views a bit broader than what is normally described as the ‘moral’. In addition to our notions and reactions on such issues as justice and the respect of other people’s life, well-being, and dignity, I want also to look at our sense of what underlies our own dignity, or questions about what makes our lives meaningful or fulfilling. These might be classed as moral questions on some broad definition. . . . They concern . . . what makes life worth living. (Taylor 1989, p. 4)

So, moral space comprises not only questions concerning our obligations towards others but also questions of how to live a full life and of how to live with dignity. Taylor sees these as inescapable, existential questions that human beings necessarily face. Human beings cannot but try to find their orientation in the space constituted by these questions. Processes of orienting in moral space are existential processes that may be understood as processes of searching for meaning in life (Schuhmann and van der Geugten 2017).

Generally, in order to find our orientation we need orienting frameworks by which we can judge where we are and in what direction we should go. In moral space, these frameworks are made up of what Taylor (1989) calls ‘visions of the good’—visions of a life worth living—that represent convincing answers to questions of how to live. These visions of the good form “the horizon within which we know where we stand” (Taylor 1989, p. 29) and function as orientation points that indicate in which direction to go. Taylor stresses that such visions involve what he calls ‘strong evaluation.’ This means that they do not depend on “our own desires, inclinations, or choices, but rather stand independent of these and offer standards by which they can be judged” (Taylor 1989, p. 4). According to Taylor, the aspiration to move towards and be close to strongly valued goods—to feel that these goods are well integrated in our life—is a fundamental human craving. He even argues that “we cannot do without an orientation to the good” (Taylor 1989, p. 47). We need an orientation towards the good in order to experience our lives as meaningful. Furthermore, an orientation to the good is constitutive of our identity and agency. Taylor explains this close connection between identity, agency, and orientation in moral space as follows:

My identity is defined by the commitments and identifications which provide the frame or horizon within which I can try to determine from case to case what is good, or valuable, or what ought to be done, or what I endorse or oppose. (Taylor 1989, p. 27)

Understanding human existence in terms of orientation processes in moral space seems a suitable starting point for developing a view of pastoral care as existential work that allows for both religious and nonreligious interpretations. Generally, pastoral caregivers meet with people in situations that may be understood in terms of disorientation in moral space. People who face loss, illness, death, incarceration, or violence may feel that their sense of being close to or moving towards ‘the good’—towards a life worth living or a full life—is challenged or even shattered. They face existential questions that they cannot deal with and fail to experience a sense of meaning in life (Schuhmann and van der Geugten 2017). Sometimes they explicitly describe their situation in terms of disorientation: they don’t know where to go or how to go on, they feel lost, or they feel they are wandering in the dark. According to Taylor (1989), due to the temporal dimension of our lives, experiencing disorientation is not something exceptional. As we live in time, “our place relative to the good. .. is constantly challenged by the new events of our lives, as well as constantly under potential revision” (Taylor 1989, p. 47). Generally, we only experience minor disorientation, and we manage to reorient in a habitual way that does not involve reflection. However, life events may also challenge our orientation to the good in a more fundamental way. People who seek support from pastoral caregivers generally feel disoriented in a deep and unsettling way and do not easily or quickly manage to reorient. The central concern of pastoral caregivers may then be understood as supporting people’s search for (re)orientation in moral space (Schuhmann and van der Geugten 2017).

This view of pastoral care as work in moral space agrees with the view of pastoral care as engaging with the existential struggles of people and their search for meaning (Doehring 2015; Thorstenson 2012). It also agrees with the widespread view of pastoral care as dealing with people’s spiritual issues. In Taylor’s (1989, 2007) broad conception of morality, moral and spiritual issues are entangled. Finding our orientation in moral space entails managing to “make sense of our lives spiritually” (Taylor 1989, p. 18). Understanding pastoral care in terms of orientation processes also fits in with the view of pastoral care as engaging with “embodied lived theologies” (Doehring 2015, p. 4). The metaphor of orientation emphasizes the fundamental role of corporeality in human existence. According to Merleau-Ponty (1960), our orientation in the world depends on the perspective determined by our bodily situation. The way in which things appear to us and obtain prereflective meaning depends on our bodily orientation. This suggests that orientation processes in moral space have a bodily dimension and that pastoral care, understood in terms of engaging with orientation processes, involves approaching people as corporeal as well as spiritual beings.

## The ‘pastoral quality’ of work in moral space

Understanding pastoral care as work in moral space implies that pastoral caregivers engage with people’s attempts to orient towards a good life—a life worth living or a full life. Here, ‘a good life’ involves strong evaluation and refers to what is “of crucial importance, or of fundamental value” (Taylor 1989, p. 42) to people and is constitutive of their identity. This description of pastoral care as engaging with people’s attempts to orient towards crucial goods does not fully capture the unique quality of pastoral care because it applies to other professions as well. For instance, social workers, physicians, and psychologists all somehow help people to (re)orient towards goods that may be of crucial importance to them, such as (mental) health or well-being. What, then, is the characteristic pastoral quality of pastoral caregivers’ engagement with people’s orientation processes in moral space?

For a start, pastoral caregivers do not seem to support orientation towards an obvious characteristic ‘pastoral good’ in a way similar to how physicians support orientation towards physical health or psychologists towards psychological health. In fact, pastoral caregivers do not take a preconceived notion of visions of the good that people orient towards as the fixed starting point of their practice. Work in moral space is work in the domain of meaning regarding which visions of the good are of fundamental value to someone, at this point in his or her life, given the circumstances (Schuhmann and van der Geugten 2017). Pastoral caregivers would not presume to know the answer to this question but sensitively and attentively support people in searching for and figuring out their own answers (Doehring 2015; Lynch 2002). In order to understand the distinctive role of pastoral caregivers, instead of attempting to identify characteristic pastoral goods that pastoral caregivers would support people orienting towards, we might also focus on the kind of situations in which the specific character of pastoral care most obviously comes to the fore. The role of pastoral caregivers is probably most salient in ‘ultimate’ situations—situations of severe disorientation in which our usual visions of the good lose their believability (Schuhmann and van der Geugten 2017). Ultimate situations may involve experiences of wonder or awe that do not fit in with our usual visions of the good and which render these visions of the good inadequate or irrelevant. Ultimate situations may also be desperate situations; we may fall fatally ill with no prospect of regaining health, or we may be the victim of violence and feel that there is no good left in the world. In these situations, we cannot simply reorient towards our original visions of the good. We need to search for new visions of the good that have not lost all believability in these desperate or awe-inspiring situations. Pastoral care seems specifically salient in situations in which people face intense suffering and evil so that the search for believable visions of the good seems senseless and hopeless.

This suggests that pastoral caregivers, independent of their tradition, do not promote a fixed vision of the good but rather represent the possibility of somehow, eventually, connecting to some good that is not rendered utterly meaningless by suffering and evil. In the context of chaplaincy, it is said that a distinctive characteristic of pastoral caregivers is that “they represent and manifest claims about the nature of reality” (Cobb et al. 2015, p. 2). The precise content of these claims depends on the specific (and often religious) tradition represented by the pastoral caregiver. In religious contexts, goods that are not undone by suffering and evil are related to God. Here, pastoral caregivers represent the possibility of connecting to God, even in the most desperate situations. However, the idea of a good that is not rendered meaningless by suffering and evil is not restricted to religious traditions only. For instance, existentialist thinker Frankl (1959), on the basis of his experiences in a concentration camp, developed logotherapy around the claim that “we may also find meaning in life even when confronted with a hopeless situation, when facing a fate that cannot be changed” (p. 112). From a philosophical viewpoint, Murdoch (1970) explores the question of how to connect “a clear-eyed contemplation of the misery and evil of the world with a sense of an uncorrupted good” (p. 59). She argues that, in a world without God, we need to retain an idea of the Good as the focus of moral life. We will argue that these ideas are helpful for theoretically underpinning the specifically pastoral quality of pastoral care in a secular age.

## Murdoch on good and transcendence

Like Taylor (1989), Murdoch (1970) develops a broad perspective on morality. She states that morality not only concerns the question of how to choose well at certain moments in our life but touches upon our lives as a whole: “The area of morals can now be seen as covering the whole of our mode of living and the quality of our relations with the world” (Murdoch 1970, p. 95). Human beings are “spiritual creatures, attracted by excellence and made for the Good” (Murdoch 1970, p. 100). So, again like Taylor, Murdoch associates spirituality with striving for the good and stresses that not just any good we strive for in life can be seen as the focus of a spiritual quest. Where Taylor speaks about incomparably higher goods or ultimate goods that involve strong evaluation, Murdoch uses the overarching concept of the Good. Rather than referring to specific ultimate goods, the Good seems to refer to a specific quality that ultimate goods may or may not have.

Murdoch’s (1970) concept of the Good is maybe best understood through her idea of transcendence. Transcendence means in the first place transcendence of ego: “Goodness is connected with the attempt to see the unself, to see and to respond to the real world” (p. 91). Murdoch departs from a view of people as naturally selfish; the moral task that human beings face in life is to shift from selfishness to unselfishness. This is not an easy task since we are inclined to focus our attention on our “fat relentless ego” (p. 51). Moral development is the difficult process of purifying ourselves by turning our attention away from ourselves. So, moral development involves transcending our ego and its selfish inclinations.

Second, Murdoch (1970) associates transcendence with realism. The movement beyond the self is at the same time a movement towards reality. Murdoch uses the metaphor of vision to elucidate the connection between unselfishness and realism. She argues that looking at reality as it is requires a loving, compassionate gaze that is not obscured by our private and often selfish fantasies. According to Murdoch, “Human beings cannot bear much reality” (p. 62) and, rather than connecting with reality, escape from it by retiring into their private, consolatory fantasy worlds. It requires discipline and dedication to turn away from these comforting fantasies in order to look at reality, which is why Murdoch understands realism as a “moral achievement” (p. 64). Looking at reality involves looking at the reality of human suffering and “the real existence of evil: cynicism, cruelty, indifference to suffering” (p. 95). It also involves looking at the reality of “the existence of other people and their claims” (p. 57).

Third, the Good is transcendent in the sense that it is an orientation point that always remains distant. It shows us directions in which to go, yet “it lies always beyond, and it is from this beyond that it exercises its *authority*” (Murdoch 1970, p. 61). Our attempts to see reality as it is are never exhaustive due to psychological limitations connected with our selfish nature and due to the inexhaustible complexity of reality. In other words, the Good refers to a perfection that human beings can approach but never achieve. In this regard, Murdoch speaks about the nonrepresentability or indefinability of the Good and about its mysteriousness. The Good remains a mystery as reality eventually remains mysterious to us. Here, she uses Plato’s metaphor of the sun. We cannot look directly at the sun: “We do not and probably cannot know, conceptualize, what it is like in the centre” (p. 97). We can attempt to look at things in the light of the Good, but we cannot look at the Good itself. In particular, Murdoch’s account of the Good and transcendence involves a view of human beings as necessarily imperfect and fallible: “Good is mysterious because of human frailty” (p. 96).

Murdoch’s notion of transcendence makes sense in both religious and nonreligious accounts of morality and spirituality. Taylor (2007) states something similar:

In our religious lives we are responding to a transcendent reality. We *all* have some sense of this, which emerges in our identifying and recognizing some mode of what I have called fullness, and seeking to attain it. (p. 768, emphasis added)

Psychologist Freeman (2014a, b) uses Murdoch’s ideas on transcendence when presenting transcendence as an essential aspect of selfhood and self-realisation of all people. Recently, he and other authors have called for a reappraisal of transcendence as a central concept in psychology (Freeman 2014b; Richardson 2014; Slife and Richardson 2014). They argue that “any viable concept of the self is always ‘more than mere self’” (Slife and Richardson 2014, p. 321). Freeman (2014a) relates the transcendent dimension of selfhood to experiences of human or nonhuman ‘otherness,’ of the “felt reality of the Other” (p. 154). He sees the Other as the primary source of both existential nourishment and ethical enrichment. Building on the work of Murdoch, Freeman emphasizes the ethical dimension of transcendence related to acknowledging the Other as fundamentally mysterious, “the Other as a reality whose meaning transcends me” (p. 156).

## Representing the good in practices of pastoral care

Following Murdoch’s ideas on Good as transcendent, we propose to understand the specifically pastoral quality of pastoral care as ‘representing the Good.’ We hope that this offers a step towards a shared understanding of ‘pastoral’ that makes sense in different religions and worldviews. We realize that the expression ‘representing the Good’ may easily be misunderstood when ‘the Good’ is understood in a simplified way, without reference to Murdoch’s subtle and complex ideas about the Good. We therefore emphasize that ‘representing the Good’ should not be confused with imposing a specific vision of the good on others or claiming that one is Good oneself. The Good, as Murdoch understands it, is something we can only strive for but never arrive at, something which eventually remains mysterious. We cannot grasp the Good or be ‘completely good,’ let alone prescribe the Good to others. The Good is not associated with invulnerability but with human vulnerability and imperfection and with what Nussbaum (2001) calls ‘the fragility of goodness.’ In particular, representing the Good does not agree with perspectives on pastoral care that emphasize a kerygmatic orientation in which the proclamation of one specific vision of the good is the central concern. Representing the Good is rather to be understood as representing the faith that some good remains believable in ultimate situations, that it makes sense to keep searching for such a good even though it remains to some extent mysterious. This is in line with the widespread view of pastoral caregivers as ‘agents of hope’ (Capps 1995; Drescher and Foy 2010; Nolan 2011). So, whether or not pastoral caregivers work from a religious inspiration, their work involves a kind of faith: faith that it makes sense not to give up on goodness even though goodness is fragile. Freeman (2014a) also points to the connection between transcendence and ‘a kind of faith’: “To invoke realities beyond the perimeter of the self is inevitably to bring *faith*, of a sort, into the picture” (p. 154).

Let us now explore how the idea of ‘representing the Good’ translates to pastoral care practice and how it fits in with existing perspectives on pastoral care. We will do so by consecutively zooming in on three aspects of pastoral care practice: the pastoral relationship with clients, the pastoral response to stories of clients, and the pastoral role in organizations and society at large. With respect to all three aspects, Freeman’s (2014a, b) conception of transcendence as acknowledging the mysteriousness of the—human or nonhuman—Other is helpful. Concerning the pastoral relationship, representing the Good means recognizing the mysteriousness of clients as Others. This underlines the relevance of Levinas’s work to understanding the pastoral relationship (Doehring 2015; Dueck and Goodman 2007; Dueck and Parsons 2007; Lynch 2002). Doehring (2015), for instance, explains, “According to Levinas we choose life when we become open to the mystery of the other” (p. 47). Murdoch’s Good, however, is not just mysterious and distant but also involves a sustained effort of moving towards it by compassionately looking at reality. So, representing the Good entails not only recognizing the alterity of clients but also making a sustained effort to connect with them. Building on the work of Levinas, among others, Butler (2005) offers an ethical perspective on the relational dynamics of recognizing the irreducible alterity of others and simultaneously attempting to understand them. She proposes rethinking recognition as a process of desiring to know the other while keeping this desire alive at all times. In this perspective, an ethical address of the other consists of “asking the question ‘Who are you?’ and continuing to ask it without any expectation of a full or final answer” (p. 43). Butler argues that in this way we “let the other live” (p. 43); an ethical address sustains and promotes life. Butler’s work seems promising for theoretically underpinning the pastoral relationship in practices of representing the Good.

With respect to the pastoral response to stories of clients, recognizing the mysteriousness of the Other refers in the first place to the otherness of the client. As representatives of the Good, pastoral caregivers look with compassion and love at their clients’ reality and thus listen carefully to their stories. Furthermore, they realize that their attempts to see the reality of their clients are never exhaustive, that there is always more to see, to hear, and to know about them. So, they need to attune their response to the particularity of a client’s story instead of giving ready-made answers and at the same time keep listening for what has not been said so far and also for what cannot be said and remains unarticulated. So, practices of representing the Good are client-centered; the visions of the good of the client are the primary focus of attention, not those of the pastoral caregiver. This applies in particular when the client’s story involves severe suffering and tragedy. As representatives of the Good, pastoral caregivers cannot respond to suffering and tragedy by moving away from it, for instance by offering consoling stories that cover up the other’s suffering. Their response needs to be embedded in efforts to genuinely engage with the suffering other. Second, recognizing the mysteriousness of the Other when responding to a client also refers to ‘others’ involved in the life of the client. Representing the Good involves looking with love and compassion at all of reality, not just at the reality of a particular client. So, client-centeredness does not imply that pastoral caregivers take an ‘anything goes’ stance with respect to the visions of the good that people commit themselves to—even when people see these as spiritual or sacred visions. Taylor (1989) remarks that culturally sanctified goods may well be “incompatible with justice or human dignity” (p. 66). According to Pargament (2007), “‘Spirituality’ is not a synonym for ‘goodness’” (p. 129). And Lynch (2012) points out that “sacred forms. .. have the capacity to legitimate oppressive social orders, violence, and the breach of basic human rights of freedom and well-being” (p. 114). Pastoral caregivers would certainly not support visions of the good that involve violence, oppression, or injustice. In particular, when clients reduce others to objects in self-centered stories, representing the Good includes taking the ethical stance that these others—like the client—are unique beings who are too complex to grasp. It is important to emphasize this ethical aspect of pastoral care because people not only may and do suffer as a result of actions of others but themselves may and do inflict suffering on others, as well. For instance, pastoral caregivers working with prisoners regularly need to respond to stories in which damage has been inflicted both on and by clients (Schuhmann 2015).

Finally, understanding pastoral care in terms of representing the Good highlights the role of pastoral caregivers in the organizations in which they work and in society at large. Taylor (1989) explains that orienting in moral space is never a purely individual process. We do not invent our own visions of the good but adopt them from our sociocultural contexts. What counts as ‘good’ depends on the context: “A vision of the good becomes available for the people of a given culture through being given expression in some manner” (Taylor 1989, p. 91). So, when pastoral caregivers support people’s search for (re)orientation in moral space, they need to address the question of which visions of the good are available to whom in a given situation. Here representing the Good implies in the first place supporting and contributing to efforts of creating and strengthening visions of the good that represent an aspiration for the Good. Practices of representing the Good include promoting dialogue and including marginalized or easily overheard voices in the dialogue. For instance, pastoral caregivers may initiate moral deliberation in the organizations in which they work. In public space, they may contribute to various current public discussions on the good life. This points to the political dimension of pastoral care that has also been emphasized by LaMothe (2014).

Second, representing the Good means taking a critical stance towards dominant visions of the good in organizations and society and challenging these visions when they include stereotyping or become prescriptive, oppressive, and dehumanizing. Similarly, Doehring (2015) describes social justice as an important goal of pastoral care. Furthermore, representing the Good implies challenging dominant visions of the good that present life as fully comprehensible and thus rule out mysteriousness, vulnerability, and fallibility. When, for instance, a specific vision of the good is presented as an exhaustive representation of the Good, or when human life is evaluated in medical, juridical, psychological, economic, or financial terms, the mystery of life disappears from view and the Good is lost. Pastoral caregivers represent the view that “no completely adequate and comprehensive worldview is possible, and behind all the pretense to absolute and ultimate knowledge, the sense for the irrationality of human life, for the fact that it is unlimitable, remains” (Geertz 1973, p. 139). Following Geertz (1973), we might conclude that recognizing “the hollowness of human pretensions to religious or moral infallibility is perhaps the surest sign of spiritual maturity” (p. 140).

## Brief recapitulation and concluding remarks

In this paper, we have used the philosophical work of Taylor (1989, 2007) and Murdoch (1970) to address the question of how to conceptually understand pastoral care in a secular age. As explained in the introduction, the challenge here is to come to an understanding of pastoral care that includes practitioners of different worldview traditions and that pictures pastoral care as relevant for people of “all religions and none.” Following the work of Taylor, we have developed a view of pastoral care as work in ‘moral space.’ In this view, pastoral care is in the first place existential work since, as Taylor emphasizes, orienting in moral space is part and parcel of people’s existence. It is also spiritual work as the question of what people perceive as ultimately good or sacred is a central issue. All human beings may, at some point in their lives, get disoriented and need spiritual care when attempting to reorient. As different health care professionals claim authority to provide this care, pastoral caregivers face the challenge of explaining the unique quality of the spiritual care that they provide. Following the richness of Murdoch’s ideas on Good as transcendent, we conceptualized this unique quality as ‘representing the Good.’ The notion of representation indicates that not all spiritual care counts as pastoral care. Pastoral caregivers represent a view of reality as ultimately mysterious and the faith that it makes sense not to give up on goodness. This requires of pastoral caregivers not only that they have knowledge of the diverse traditions of the good that are of importance to potential clients but also that they keep exploring their own position with respect to existing traditions and keep fostering a living relationship with the Good that involves an acute awareness of ‘the fragility of goodness.’

In our conceptual explorations, we did not aim for a completely new view of pastoral care but for a perspective that contributes to an inclusive theoretical underpinning of existing pastoral care practice in all its variety. Throughout the paper, we have indicated connections between our perspective and ideas in the existing literature on pastoral care. In particular, the perspective that we have developed connects to the idea that meaning-making in the face of existential struggles should play a central role in the contemporary understanding of pastoral care (Doehring 2015; Thorstenson 2012). Given the close connection between orienting processes in moral space and processes of searching for meaning in life (Schuhmann and van der Geugten 2017), we would rather use the notion of searching for meaning in life than the term meaning-making. Speaking of searching for meaning in life highlights the existential and sociocultural (and not just the psychological) dimensions of processes of searching for meaning. Whereas the term meaning-making suggests that meaning can be actively created, the notion of searching for meaning in life emphasizes the vulnerability of human beings and the role of receptivity in and the fallibility of our attempts to orient in life.

Obviously, in order to legitimize pastoral care in secular contexts, an inclusive theoretical understanding of pastoral care is not sufficient. For health care chaplains in particular, there is an increasing demand for legitimation through research evidence: “In order to build the case that we are productive members of the health care team, we have to provide evidence for the beneficial effects of the care we provide” (Fitchett 2011, p. 4). Such research requires a sound theoretical basis: “From a professional perspective, chaplains need to be able to define what is unique about their input” (Snowden et al. 2012, p. 3). While we hope that this description contributes to an understanding of the unique input of pastoral caregivers, we also want to point out that, if recognition of mysteriousness lies at the heart of pastoral care, empirical research on pastoral care practices requires a critical view of (dominant) research strategies that rule out mysteriousness.

The perspective on pastoral care that has been developed in this article highlights not only the existential and spiritual dimensions of pastoral care but also its ethical and political dimensions. Practices of representing the Good have an ethical dimension because striving for the Good involves an effort to look with love and compassion at human—and nonhuman—‘otherness.’ Representing the Good also involves addressing the political question of which visions of the good are available to whom. In our globalizing, complex world, the ethical and political dimensions of pastoral care seem to deserve special attention. These days, it is painfully clear that people’s opportunities for voicing and acting upon their aspirations for a good life are far from equal (Appadurai 2004). Furthermore, because people are interrelated on a global scale, their attempts of pursuing the good interfere in complex ways that inevitably at times lead to tension and conflict (Gergen 2009; Hermans and Hermans-Konopka 2010). In particular, processes of orienting in moral space are globally intertwined; one person’s quest for a strongly valued good may both thwart and be thwarted by others’ quests for what they perceive as strongly valued goods (Schuhmann 2016). If pastoral care is conceived of in terms of representing the Good, then pastoral caregivers need to take this into account—even in situations where they work with just one client—and cannot ignore the ethical and particularly the political aspects of their work.

When ‘pastoral’ and ‘secular’ are understood as opposites, pastoral care may seem an obsolete phenomenon in a secular age. When, however, we go along with Taylor’s (2007) idea that people in a secular age still respond to transcendent reality by attempting to orient towards ultimate goods, then pastoral care, in the sense of supporting these attempts, seems just as relevant today as it was in the presecular age. In a secular age, the self-evidence of religious orienting frameworks is lost. According to Taylor (1989), we live in a world in which there is “no ultimately believable framework” (p. 17) that guides us through moral space. In a globalizing world, we come into contact with “myriad traditions of the good” (Gergen 2009, p. 358) springing from myriad different relational contexts. All this does not facilitate the orientation processes of contemporary people. In a way, we might say that disorientation in moral space is a condition of our time. Similarly, Gilligan (2014) argues that “the search for moral truth” (p. 101) has become a complex affair in a postmodern age. Rethinking pastoral care as a practice of supporting people, organizations, and societies in dealing with the complex task of searching for moral truth reveals the enormous potential of pastoral care in a secular age.
